# P2Y_2_-P2X7 receptors cross-talk in primed mesenteric endothelial cells upregulates NF-κB signaling favoring mononuclear cell adhesion in schistosomiasis

**DOI:** 10.3389/fimmu.2023.1328897

**Published:** 2024-01-04

**Authors:** Nathália Ferreira Oliveira, Matheus Macedo L. V. Monteiro, Nathália Santos Mainieri, Augusto Shuiti Tamura, Letícia Massimo Pereira, Leticia Diniz Crepaldi, Robson Coutinho-Silva, Luiz Eduardo Baggio Savio, Claudia Lucia Martins Silva

**Affiliations:** ^1^ Laboratório de Farmacologia Bioquímica e Molecular, Universidade Federal do Rio de Janeiro, Rio de Janeiro, Brazil; ^2^ Laboratório de Imunofisiologia, Universidade Federal do Rio de Janeiro, Rio de Janeiro, Brazil

**Keywords:** endothelial cell, schistosomiasis, P2Y2 receptor, P2X7 receptor, mesenteric vessels, inflammation, cell signaling, purinergic signaling

## Abstract

Schistosomiasis is an intravascular infectious disease that impacts over 200 million people globally. In its chronic stage, it leads to mesenteric inflammation with significant involvement of monocytes/macrophages. Endothelial cells lining the vessel lumens play a crucial role, and mount of evidence links this disease to a downregulation of endoprotective cell signaling favoring a primed and proinflammatory endothelial cell phenotype and therefore the loss of immunovascular homeostasis. One hallmark of infectious and inflammatory conditions is the release of nucleotides into the extracellular milieu, which, in turn, act as innate messengers, activating purinergic receptors and triggering cell-to-cell communication. ATP influences the progression of various diseases through P2X and P2Y purinergic receptor subtypes. Among these receptors, P2Y_2_ (P2Y_2_R) and P2X7 (P2X7R) receptors stand out, known for their roles in inflammation. However, their specific role in schistosomiasis has remained largely unexplored. Therefore, we hypothesized that endothelial P2Y_2_R and P2X7R could contribute to monocyte adhesion to mesenteric endothelial cells in schistosomiasis. Using a preclinical murine model of schistosomiasis associated with endothelial dysfunction and age-matched control mice, we showed that endothelial P2Y_2_R and P2X7R activation increased monocyte adhesion to cultured primary endothelial cells in both groups. However, a distinct upregulation of endothelial P2Y_2_R-driven canonical Ca^2+^ signaling was observed in the infected group, amplifying adhesion. In the control group, the coactivation of endothelial P2Y_2_R and P2X7R did not alter the maximal monocyte adhesion induced by each receptor individually. However, in the infected group, this coactivation induced a distinct upregulation of P2Y_2_R-P2X7R-driven canonical signaling, IL-1β release, and VCAM-1 expression, with underlying mechanisms involving inflammasome and NF-κB signaling. Therefore, current data suggest that schistosomiasis alters endothelial cell P2Y_2_R/P2X7R signaling during inflammation. These discoveries advance our understanding of schistosomiasis. This intricate interplay, driven by PAMP-triggered endothelial P2Y_2_R/P2X7R cross-talk, emerges as a potential key player in the mesenteric inflammation during schistosomiasis.

## Introduction

Schistosomiasis is an intravascular infectious disease and a global leading cause of mortality. The World Health Organization (WHO) estimates that the disease affects more than 230 million people worldwide (1).

The vascular mesenteric function has clinical importance in health and disease, including inflammatory diseases (2). Within the mesenteric vessels, the contact of *Schistosoma mansoni* and eggs with endothelial cells and the recognition of their antigens (PAMPs) by host pattern recognition receptors (PRR) trigger immune responses, endothelial cell activation, liver, intestine, and mesenteric inflammation and disease progresses from acute to chronic infection, with significant contributions from monocytes/macrophages (3–7). Given the number of infected people and morbidity, there is an urgent need to discover a complementary treatment (6).

Endothelial cells line the entire vascular system, sensing and responding to the extracellular milieu with physiological or maladaptive responses such as loss of endoprotective signaling. In the mesentery, they control leukocyte transmigration to extravascular tissues and inflammation (8). Proinflammatory mediators causing vascular remodeling increase chronic leakage (9), an inflammatory trait observed in schistosomiasis (2, 6, 10).

Parasite migration induces the release of damage-associated molecular patterns (DAMP) such as ATP that modulate host immune responses (4). In pathological conditions, extracellular ATP acts as a versatile extracellular messenger, conveying important information across a broad spectrum of cell types and tissues (11). Extracellular ATP conveys intracellular signaling through ionotropic (P2X) or 7TM/G protein-coupled purinergic receptors (P2YR) (12–14). The canonical signaling of P2Y_2_ (P2Y_2_R) and P2X7 receptors involves the increase of intracellular Ca^2+^, inflammasome activation, and interleukin (IL)-1β release, a proinflammatory cytokine (14–17).

A typical finding in chronic schistosomiasis is portal-mesenteric inflammation (18–20), which depends on the proinflammatory (i.e., dysfunctional) phenotype of endothelial cells. The endothelial P2Y_2_R has been studied in some vascular injury models (21–23), but it has yet to be explored within the context of intravascular infectious diseases like schistosomiasis. Recently, it was shown that the P2YR-dependent Ca^2+^ signalosome is altered during endothelial dysfunction, shedding light on its potential importance (17). Conversely, the endothelial role of P2X7R is still not fully clear. In the present study, we investigated the contribution of endothelial P2Y_2_R and P2X7R to monocyte adhesion on endothelial cells primed by schistosomiasis. Our findings in the infected group unveiled a distinct positive cooperation between endothelial P2Y_2_R and P2X7 signaling, favoring IL-1β release and vascular cell adhesion molecule-1 (VCAM-1)-mediated monocyte adhesion to mesenteric endothelial cells, which might be critical to schistosomiasis mesenteric inflammation.

## Materials and methods

### Animals and infection with *S. mansoni*

Male Swiss mice were obtained from the animal facilities of the Biomedical Science Institute (Federal University of Rio de Janeiro, Brazil). All procedures using animals were carried out in compliance with the ethical standards of our institution (Ethics Committee of the Federal University of Rio de Janeiro (CEUA), approved under the licenses A1/19-048-16 and 124/22, and with the recommendations of both the National Council on Experimental Animal Control (CONCEA, Brazil) and the National Institutes of Health guide for the care and use of Laboratory animals (USA). Animals were housed in a temperature-controlled room (22°C) under a light/dark cycle of 12/12 h and had access to water and food *ad libitum*.

Animals (7–10 days old) were infected percutaneously with ~80 cercariae of both genders (BH strain; obtained from infected *Biomphalaria glabrata* snails) for 8 min, as previously described (10). Animals were used after 70–80 days of infection. Age-matched control male Swiss mice were used for comparison with infected mice. Animals were anesthetized (ketamine at 75 mg/kg body weight and xylazine at 10 mg/kg body weight, ip) and euthanized by cervical dislocation. All efforts were made to minimize animal suffering, and the number of animals used was associated with valid statistical evaluation.

### Primary endothelial cell culture

Mesenteric endothelial cells were obtained from Swiss mice (uninfected or control) and *S. mansoni*-infected mice. Briefly, after anesthesia (as detailed in **Animals and infection with *S. mansoni***) and euthanasia, mesenteric vessels were carefully removed, cut into small pieces, and covered with Dulbecco’s modified Eagle’s medium (DMEM) supplemented with 20% heat-inactivated fetal bovine serum (FBS, Invitrogen, USA), NaHCO_3_ (44 mM), glucose (5.5 mM), antibiotics (penicillin/streptomycin (1% v/v)) and 1% Glutamax (pH 7.4) (Gibco, USA). The plates were kept at 37°C and 5% CO_2_. After 72 h, the explants were removed, and the medium was replaced every 48 h until ~90% cell confluence, which usually takes 10–12 days (10). Cells were then washed with 1 mL of Balanced Salt Solution (BSS) solution (NaCl 137 mM, KCl 5.36 mM, Na_2_HPO_4_ 1.08 mM, KH_2_PO_4_ 1.1 mM, and glucose 6.1 mM), pH 7.4. After 5 min, the BSS solution was removed, and the cells were treated for 5 min with 100 µL of trypsin solution (0.25% in BSS). The reaction was stopped by the addition of 1 mL DMEM supplemented with 20% FBS. Then, the cell suspension was collected and centrifuged for 7 min (200×*g* at 4°C), and the pellet was resuspended in 1 mL DMEM. Cells were counted using Trypan blue exclusion dye (viability ≥ 95%) and plated (10^4^ cells/well in a 96-well plate or 2.5 × 10^4^ cells/well in a 24-well plate) for further experiments. The identity of mesenteric endothelial cells from both groups was confirmed by flow cytometry (Accuri^®^, BD Bioscience, USA) using a fluorescent primary monoclonal antibody against platelet-endothelial cell adhesion molecule-1 (PECAM-1, phycoerythrin rat antimouse CD31; MEC13.3; 1:150, BD Bioscience, USA) and by optical microscopy (Olympus IX71 inverted microscope; ×100 magnification) (10).

### Mononuclear cell adhesion assay

Cultured endothelial cells of both groups (first passage) were plated for a leukocyte adhesion assay in a 96-well plate. After 48 h, confluent endothelial cells were stimulated with 1–300 µM UTP (5 h), 500 µM ATP (10 min), IL-1β (5 h), or vehicle (basal) for 5 h, in the absence or presence of the P2Y_2_R antagonist suramin 50 µM, selective P2Y_2_R antagonist ARC118925XX 10 µM, selective P2X7R antagonist A740003 50 nM, anti-VCAM-1 (1:50) or anti-intercellular adhesion molecule (ICAM-1; 1:50) antibodies, ectonucleotidase inhibitor (ARL 67156) 100 µM, phospholipase C (PLC) inhibitor (U73122) 1 µM, intracellular Ca^2+^ chelator BAPTA-AM 3 µM (Molecular Probes, USA), caspase inhibitor (Z-VAD-FMK 20 µM), or NF-κB inhibitor (pyrrolidine dithiocarbamate (PDTC) 3 µM) added 30 min before. Endothelial cells were then washed and coincubated with mononuclear cells (10^4^/well) isolated from peripheral blood (control and infected groups) by cardiac puncture under anesthesia using the Ficoll–Paque density gradient. After 30 min of coincubation, the wells were washed to remove non-adhered mononuclear cells. Subsequently, four fields/wells were randomly chosen and imaged using an optical microscopy Olympus IX71 (×400 magnification), to determine the number of adherent cells (10). Data were obtained from at least three different cultures for each group and condition and expressed as mean and SEM.

### Intracellular [Ca^2+^]_i_ measurement

First passage endothelial cells from both groups (10^5^ cells/plate) were plated onto glass-bottom plates (Mat Tek Life Sciences, USA) and kept (48 h) on complete cell growth medium, 37°C, 5% CO_2_. Next, cells were washed with buffered physiological solution (in mM: NaCl, 140; KCl, 5; MgCl_2_, 1; CaCl_2_, 2; glucose, 5; and HEPES, 5, pH 7.4) and incubated with 0.04% Pluronic^®^ F-127 and fura-2 AM (Molecular Probes, USA; 2.5 μM, 40 min, 37°C, 5% CO_2_) in the dark. After loading, cells were washed, transferred to a perfused chamber (P-5 Platform, Warner Instruments, Hamden, CT, USA; 200 μL), continuously perfused with physiological solution, and stimulated with 100 µM ATP or vehicle. Cells were exposed to alternate cycles of illumination with 340 nm and 380 nm excitation wavelengths, and the emission was measured at 488 nm using a lambda DG4 illumination system (Sutter Instrument, Novato, CA, USA), a 40× objective, and a 510-nm band-pass filter (Semrock, Rochester, NY, USA). Raw data were acquired for 200 s using the MetaFluor software (Molecular Devices, Sunnyvale, CA, USA), exported to Excel, and then the fluorescence ratio (F340/F380) was used to calculate the mean peak and area under the curve (AUC) (GraphPad Prism 8.0, San Diego, CA, USA, www.graphpad.com). Each assay was performed with different cell cultures in triplicates.

### Peritoneal mononuclear cells counting

To access peritoneal leukocytes, initially 5 mL of sterile BSS were administered to the peritoneal cavity. Approximately 90% of the initial volume was collected and centrifuged (5 min, 350×*g*, 4°C), and the pellet was resuspended in 1 mL of sterile BSS. Cells were stained with Turk solution (acetic acid 1% v/v and methylene blue 1% w/v; 1:20), and the total leukocyte counting was performed in a Neubauer chamber. For the differential cell counting, samples (100 µL) containing 10^5^ cells were plated and run on a cytospin centrifuge (5 min, 240×*g*, 4°C). The slides were stained with a Panotic kit (LABORCLIN^®^) and analyzed using optical microscopy (×100) considering 100 cells/field.

### Western blotting assay

Confluent endothelial cells (6-well plates) were washed with sterile PBS and 200 μL RIPA buffer was added (1% Nonidet P40, 0.25% sodium deoxycholate, 150 mM NaCl, 1 mM EDTA, 1 mM phenylmethylsulfonyl fluoride, 1 mM sodium orthovanadate, 1 mM NaF, 10 μg/mL aprotinin, 10 μg/mL leupeptin, and 50 mM Tris-HCl, pH 7.4; Sigma-Aldrich, St. Louis, MO, USA). Cell lysates were centrifuged (8,100×*g*, 10 min, 4°C), and the protein content was determined by the Lowry method. The proteins (50 µg) were loaded into 12% SDS-PAGE gels and subjected to electrophoresis at a fixed voltage of 100 V. The proteins were transferred to a nitrocellulose membrane (Millipore, USA, 100 V) and then incubated for 1 h in Tris-buffered saline (TBS) with 5% nonfat milk. Next, the membranes were washed three times for 5 min with 1× TBS-Tween solution and incubated overnight with 1:200 rabbit anti-P2Y_2_ receptor antibody (APR-010; Alomone Labs, Israel) or 1:5,000 mouse monoclonal anti-β-actin (A1978; Sigma*-*Aldrich, USA) diluted in 5% BSA 1× TBS-Tween solution. After three washes with 1× TBS-Tween solution for 5 min, the membranes were incubated with HRP-conjugated antirabbit (1:2,000) or antimouse (1:20,000) secondary antibody for 2 h. The image was obtained with the chemiluminescent detection reagent (ECL, Sigma*-*Aldrich, USA) in the ImageQuant equipment (GE Healthcare, USA).

### RNA isolation and real-time quantitative PCR

The total RNA from the first passage cells was isolated using the ReliaPrep™ RNA Miniprep Systems kit (Promega Corporation, WI, USA) according to the manufacturer’s instructions. RNA samples were quantified, and the purity was assessed using a Nanodrop Lite spectrophotometer (Thermo Fisher Scientific, NJ, USA). The synthesis of cDNA was performed using 1 µg of total RNA using the High-Capacity Reverse Transcription Kit with RNase Inhibitor (Thermo Fisher Scientific, NJ, USA) according to the manufacturer’s instructions in a Master Cycler Gradient thermocycler (Eppendorf, Hamburg, Germany).

The real-time quantitative PCR reactions (RT-qPCR) were performed using the GoTaq^®^ qPCR Master Mix (Promega Corporation, WI, USA) in a QuantStudio 1 Real-Time PCR System (Thermo Scientific, NJ, USA). The reactions were performed in a final volume of 10 μL using 2 μL of diluted cDNA (1:10) and 300 nM of each primer for the forward and reverse of the genes. The used primer sequences are: ACTB (β-actin): TATGCCAACACAGTGCTGTCTGG, TACTCCTGCTTGCTGATCCACAT; GAPDH (Gapdh): GGTCATCCCAGAGCTGAACG, TTGCTGTTGAAGTCGCAGGA; P2Y2 (P2Y_2_R): TGACGACTCAAGACGGACAG, GTCCCCTACAGCTCCCCTAC. The relative cDNA expression was calculated using the comparative cycle threshold method. The β-actin (*ACTB*) and GAPDH genes were used as an endogenous control. The results were expressed as relative expression (2^ΔΔCt^) of the gene of interest/GAPDH.

### Immunocytochemistry

First passage endothelial cells (2.5 × 10^4^) were plated on glass coverslips (13 mm) for 24 h in complete DMEM before immunostaining. Cells were then incubated in DMEM containing 0.2% SFB at 37°C in the absence (basal) or presence of PDTC at 3 µM (30 min), UTP at 100 µM (5 h), or UTP at 100 µM (5 h) plus ATP at 500 µM (10 min). After each incubation, cells were fixed for 10 min at room temperature in 10% (v/v) paraformaldehyde with 4% sucrose diluted in sterile PBS, washed twice for 5 min with Triton X-100 0.2% diluted in PBS, and then incubated for 30 min with a blocking solution (BSA 0.1% plus 10% SFB diluted in PBS). Next, cells were washed twice for 5 min with Triton X-100 0.2% diluted in PBS and incubated with either anti-VCAM-1 monoclonal antibody (1:50, Santa Cruz Biotechnology, USA) or anti-ICAM-1 monoclonal antibody (1:50, Santa Cruz) for 1 h at room temperature. Subsequently, cells were washed twice for 5 min with Triton X-100 0.2% diluted in PBS. The coverslips were dipped in ultrapure water and further mounted on slides in the presence of 5 µL DAPI (Vectashield^®^ antifade mounting medium, Vector, USA). Analysis of cells was performed by fluorescence microscopy using an Olympus IX71 microscope equipped with CellSens software (400x magnification, Olympus America Inc, USA). The fluorescence intensity quantification was performed using ImageJ software, and data were expressed as arbitrary units (U.S. National Institutes of Health, Bethesda, MD, USA, https://imagej.net/ij/, 1997-2018).

### Quantification of IL-1β secretion

The quantification of IL-1β secretion by control and primed mesenteric endothelial cells was assayed using the supernatants of cells incubated for 10 min or 5h with vehicle (DMEM without FBS) or agonists (100 µM UTP (5 h), 500 µM ATP (10 min), 100 µM UTP (5 h) plus 500 µM ATP (10 min)) in the absence or presence of Z-VAD-FMK 20 µM added 30 min before (37°C, 5% CO_2_), and according to the ELISA Kit manufacturer’s protocol (R&D Systems, USA). Samples were read with a colorimetric plate reader (Tecan, Switzerland).

### Quantification of ROS

Confluent mesenteric endothelial cells (first passage, 24-well plate) were incubated for 1 h with 200 μL of a 0.1% Nitro Blue Tetrazolium (NBT) solution in sterile PBS at 37°C and centrifuged (600 x g; 10 min). Subsequently, the plates were washed with 400 μL of PBS and centrifuged again. NBT reacts with ROS (mainly superoxide anion), forming formazan crystals that deposit in the cells. The crystals were dissolved by adding 200 μL of 2M KOH and 200 μL of 100% DMSO. Next, the supernatant (200 µL) containing the dissolved product was transferred to a 96-well plate, and the absorbance was determined by spectrophotometry at 620 nm. Untreated cells in each group were dissociated to count cell numbers at confluency per well. The experiments were performed in quadruplicate and the absorbance was normalized by 10^3^ cells per well.

### Reagents and antibodies

UTP, ATP, Pluronic^®^ F-127, suramin, ARL67156, pyrrolidine dithiocarbamate, and NBT were obtained from Sigma-Aldrich (St. Louis, MO, USA). DMEM, USA fetal bovine serum, Glutamax^®^, and penicillin-streptomycin were obtained from Gibco (Grand Island, NY, USA). BAPTA-AM and fura-2 AM were obtained from Invitrogen (Carlsbad, CA, USA). A740003, U73122, and ARC118925XX were obtained from Tocris. Anti-VCAM-1 (CD106) and anti-ICAM-1 (CD54) monoclonal antibodies conjugated with FITC were purchased from Santa Cruz Biotechnology (USA; sc13160; sc8439). Anti-PECAM (CD31) monoclonal antibody conjugated with PE was purchased from BD Pharmingen™. Z-VAD-FMK and mouse IL-1β/IL-1F2 Immunoassay Kit ELISA were purchased from R&D Systems.

### Statistics

Data were expressed as mean and SEM. The differences between two or more groups were analyzed by the Student’s *t-*test or one-way analysis of variance (ANOVA), followed by the appropriate *post hoc* multiple comparisons test, respectively, considering *p* < 0.05 (GraphPad Prism 8.0, San Diego, CA, USA, www.graphpad.com). An illustration was created with BioRender.com.

## Results

### P2Y_2_R activation in primed endothelial cells mediates an increased monocyte adhesion

**Figure 1:**
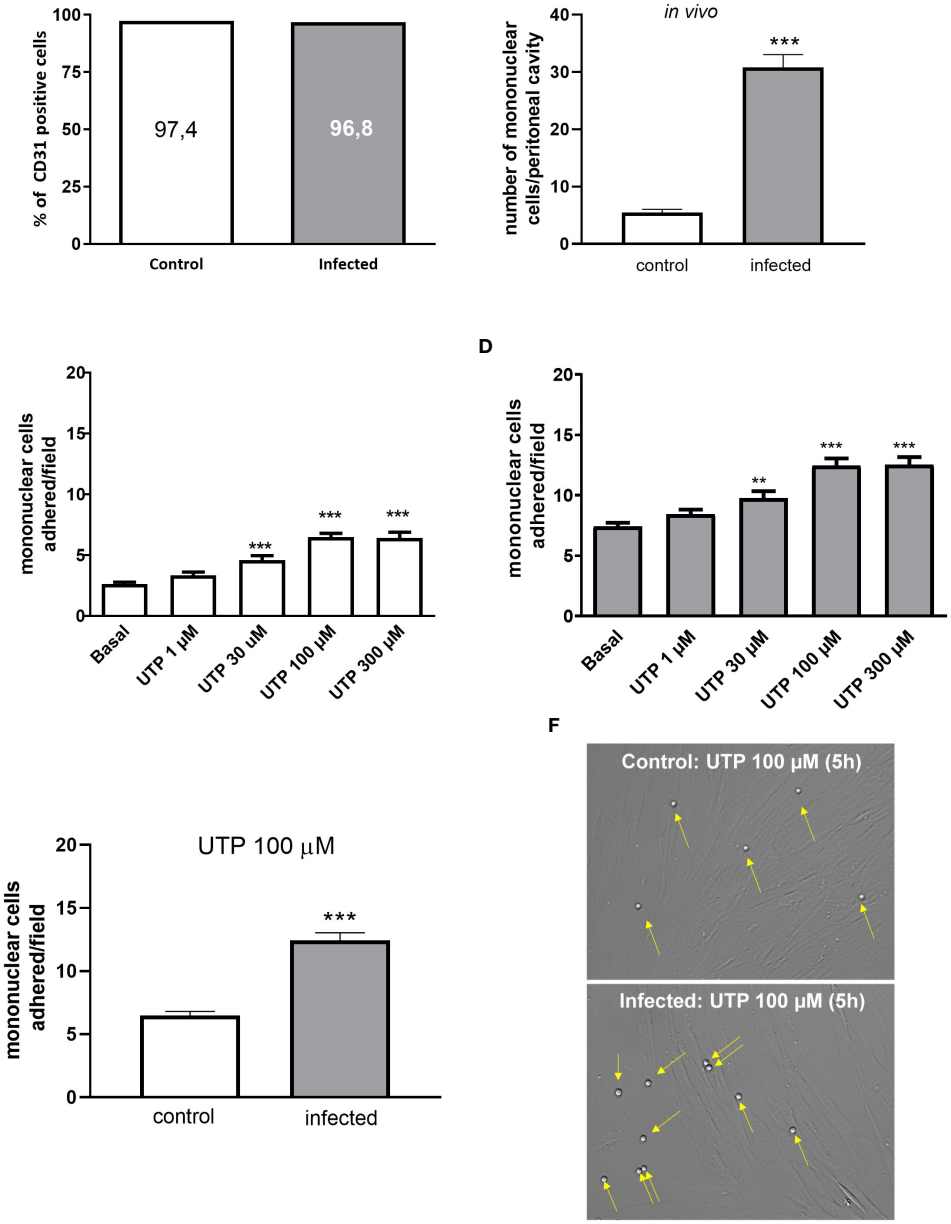
Increased UTP-induced mononuclear cells adhesion to CD31^+^ endothelial cells *in vitro.* **(A)** Cultured endothelial cells from control (white bars) and infected (gray bars) groups showed a similar percentage of cells positive for CD31. **(B)** Infected animals showed an increased number of transmigrated mononuclear cells in the peritoneal cavity *in vivo*. **(C, D)** Concentration-dependent effect of UTP inducing monocytes adhesion. **(E, F)** The number of mononuclear cells adhered to endothelial cells in response to 100 µM UTP was higher in the infected than in the control group. **(F)** Representative microscopy images (×400). Yellow arrows represent monocytes adhered to the endothelial monolayer. Data were expressed as the mean and SEM of *n* independent cultures for each condition. **(C, D)** ^**^ *p* < 0.01 and ^***^ *p* < 0.001 vs. basal (one-way ANOVA followed by Tukey’s multiple comparisons test, *n* = 3. **(B, E)** ^***^ *p* < 0.001 (Student’s *t*-test, *n* = 3).

Cultured mesenteric endothelial cells from control and *S. mansoni*-infected groups showed similar levels of CD31^+^ labeling, a marker of endothelial cells (**Figure 1A**). Accordingly with *in vivo* count of mononuclear cells in the peritoneal cavity, the transmigrated cells were higher in infected than in control mice (**Figure 1B**). Moreover, the treatment of mesenteric endothelial cells from both groups with UTP (1–300 µM, 5 h) increased mononuclear cell adhesion in a concentration-dependent manner with the maximal effect observed at 100 µM (**Figures 1C, D**). However, primed endothelial cells from the infected group showed two noteworthy traits: they displayed higher basal levels of leukocyte adhesion to endothelial cells compared to the control group, and their response to UTP stimulation resulted in even greater leukocyte adhesion (*p* < 0.05). As shown in **Figures 1E, F**, in the presence of 100 µM of UTP, the maximal adhesion of mononuclear cells was significantly higher in the infected than in the control group (*p* < 0.001, Student’s *t*-test).

**Figure 2:**
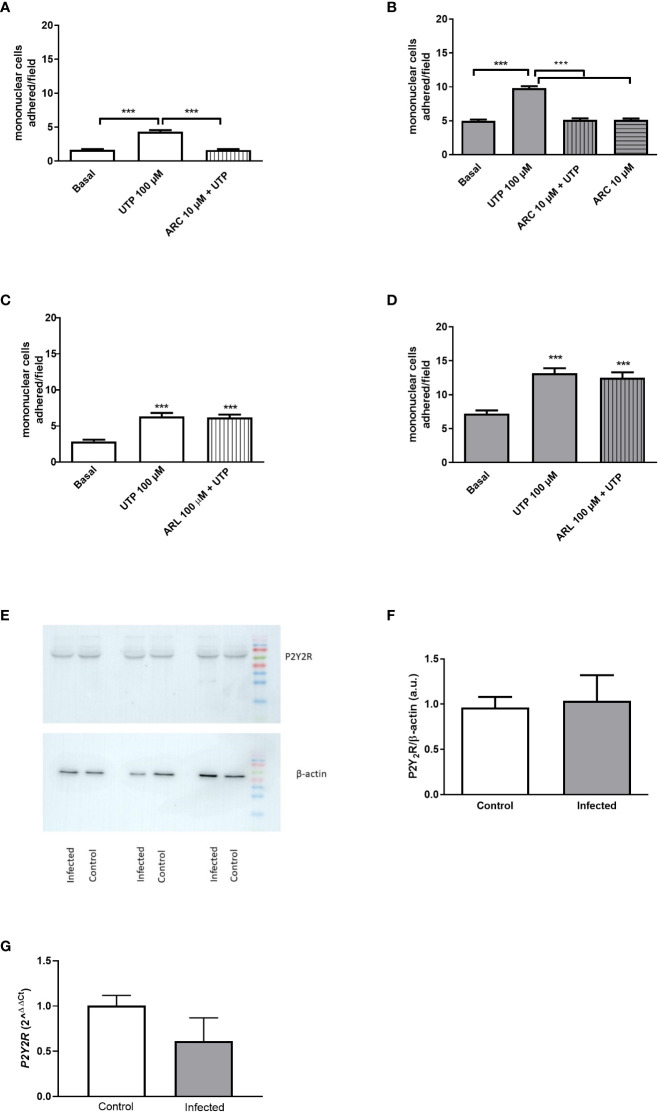
Endothelial P2Y_2_R mediates the mononuclear cell adhesion induced by UTP 100 µM, and its expression is not altered by schistosomiasis. **(A, B)** The selective P2Y_2_R antagonist ARC118925XX (10 µM; 30 min pretreatment) inhibited mononuclear cell adhesion to endothelial cells from the control (white bars) and infected (gray bars) groups. **(C, D)** The NTPDase (CD39) inhibition by ARL 67156 (100 µM; 30 min pretreatment) did not alter the UTP-induced mononuclear cell adhesion effect. ^***^ *p* < 0.001 (one-way ANOVA followed by Tukey’s multiple comparisons test, *n* = 3 **(A–D)**. **(E)** Immunoblot of P2Y_2_R in both groups. **(F)** Densitometric analysis of immunoblot data (a.u.). **(G)** P2Y_2_R mRNA levels were normalized by the endogenous GAPDH gene. Data were expressed as mean and SEM of *n* independent cultures for each condition. *p* = 0.82 and 0.12, Student’s *t*-test, *n* = 3 **(F**, **G)**.

The pretreatment of endothelial cells with the selective P2Y_2_R antagonist ARC118925XX (10 µM) blocked the UTP effect (**Figures 2A, B**). Since P2Y_2_R and P2Y_4_R (mice) are activated by UTP in the same range of concentration (24), and the later receptor is insensitive to the antagonist suramin (50 µM), we used this antagonist to rule out any contribution of P2Y_4_R to the effect of UTP. In the presence of suramin, UTP did not induce any effect in both groups (**Supplementary Figures S1A, B).**

Endothelial cells express ectonucleotidases (CD39), which hydrolyze UTP to UDP, an agonist of P2Y_6_R (11). However, as can be seen in **Figures 2C, D**, the effect of 100 µM UTP was not altered by the pre-incubation with the CD39 inhibitor (ARL 67156, 100 µM), suggesting that the endothelial effect observed is primarily mediated by P2Y_2_R promoting mononuclear cell adhesion.

The level of vascular expression of P2Y_2_R depends on the anatomical location and physiological condition (25, 26). RT-qPCR data revealed similar levels of transcripts (log 2^ΔΔCt^ = 3.3 × 10^−7^ ± 0.028 and −0.257 ± 0.107, control and infected, respectively, *p* = 0.12, Student’s *t*-test, *n* = 3-4), and Western blotting analysis revealed a single band with the expected apparent molecular mass of P2Y_2_R and similar levels of P2Y_2_R expression in endothelial cells from control and infected groups (**Figures 2E–G**).

### The upregulated P2Y_2_R canonical and VCAM-1 signaling in primed endothelial cells increase monocyte adhesion

**Figure 3:**
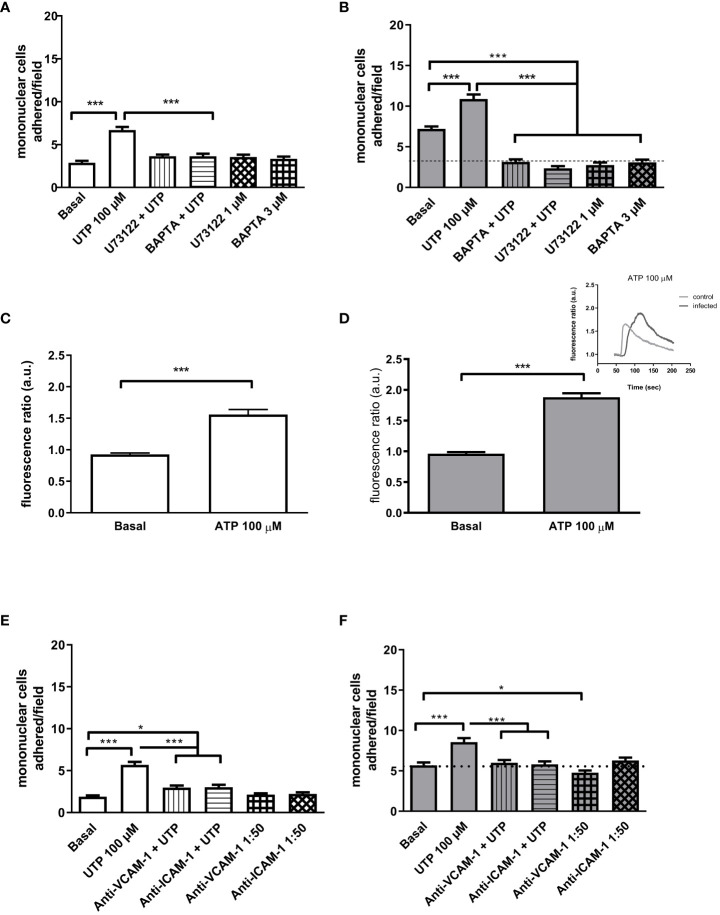
The increased role of the P2Y_2_R canonical signaling and endothelial adhesion molecules in the UTP-induced mononuclear cell adhesion in the schistosomiasis group. White bars (left), control; gray bars (right), infected group. **(A, B)** The endothelial cell pretreatment (30 min) with intracellular Ca^2+^ inhibitors U73122 (1 µM) or BAPTA-AM (3 µM) blocked the UTP-induced mononuclear cell adhesion and reduced basal adhesion values in the infected group. **(C, D)** Maximal increase of endothelial concentration of Ca^2+^ in response to 100 µM ATP (**C**, control = 1.56 a.u. ± 0.08 a.u.; **D**, infected = 1.88 a.u. ± 0.06 a.u., *p* < 0.05). Insert typical register of time-lapse variation of intracellular Ca^2+^ in endothelial cells in response to ATP. Light line, control group; dark line, infected group. **(E, F)** Endothelial cell preincubation with monoclonal VCAM-1 or ICAM-1 antibodies (30 min) blocked the UTP-induced mononuclear cell adhesion. In the infected group **(F)**, VCAM-1 antibody also reduced the basal mononuclear cell adhesion. Data were expressed as the mean and SEM of *n* independent cultures for each condition. ^***^ *p* < 0.001 (one-way ANOVA followed by Tukey’s multiple comparisons test, *n* = 3–4 **(A**, **B)**. ^***^ *p* < 0.001 (*n* = 10–18 replicates from three cultures for each condition, Student’s test) **(C**, **D)**. ^***^ *p* < 0.001, ^*^ *p* < 0.05 (one-way ANOVA followed by Tukey’s multiple comparisons test, *n* = 3) **(E**, **F)**. U73122, phospholipase C inhibitor; BAPTA-AM, Ca^2+^ chelator; anti-VCAM-1, vascular cell adhesion molecule antibody (dilution 1:50); anti-ICAM-1, intercellular cell adhesion molecule antibody (dilution 1:50).

The canonical signaling of P2Y_2_R is mainly through G_q/11_ with subsequent activation of phospholipase C (PLC) and an increase of intracellular Ca^2+^ (14). Thus, we pre-treated cells with either the PLC inhibitor U73122 (1 µM) or the intracellular Ca^2+^ chelator BAPTA-AM (3 µM). In the control group, U73122 and BAPTA blunted the UTP effect (**Figure 3A**) without altering basal values. However, in cells from the infected group, these two pharmacological tools not only prevented mononuclear cell adhesion induced by 100 µM UTP but also significantly reduced the basal values *per se* (**Figure 3B**), suggesting that primed cells show increased proadhesive intracellular Ca^2+^ signaling. In good accordance, the activation of P2Y_2_R in fura-2 loaded endothelial cells induced a higher increase of intracellular Ca^2+^ in the infected (1.88 a.u. ± 0.06 a.u.) than in the control group (1.55 a.u. ± 0.07 a.u., *p* < 0.01, Student’s *t*-test) (**Figuress 3C, D**). Also, the analysis of the mean temporal curves of intracellular Ca^2+^ showed an enhanced response in the infected group represented by a larger area under the curve than control (AUC = 228.2 and 202.8, respectively), indicating that the disease alters Ca^2+^ handling by endothelial cells. We also investigated the endothelial P2Y_2_R noncanonical signaling which links Src and VEGF receptor transactivation. However, in this case, the pharmacological inhibition of Src with SU6656 prevented the pro-adhesive effect of UTP in both groups similarly and without reducing the basal values (**Supplementary Figures S2A, B**).

In endothelial cells, the increase of intracellular Ca^2+^ favors the expression of VCAM-1 and ICAM-1. Accordingly, the antagonism of VCAM-1 and ICAM-1 inhibited the effect of UTP in both groups (**Figures 3E, F**). However, in the infected group, the antagonism of VCAM-1 also reduced slightly the basal adhesion values (**Figure 3F**, *p* < 0.05).

### P2Y_2_R and P2X7R signaling crosstalk in primed endothelial cells by schistosomiasis upregulates NF-κB signaling and monocyte adhesion

**Figure 4:**
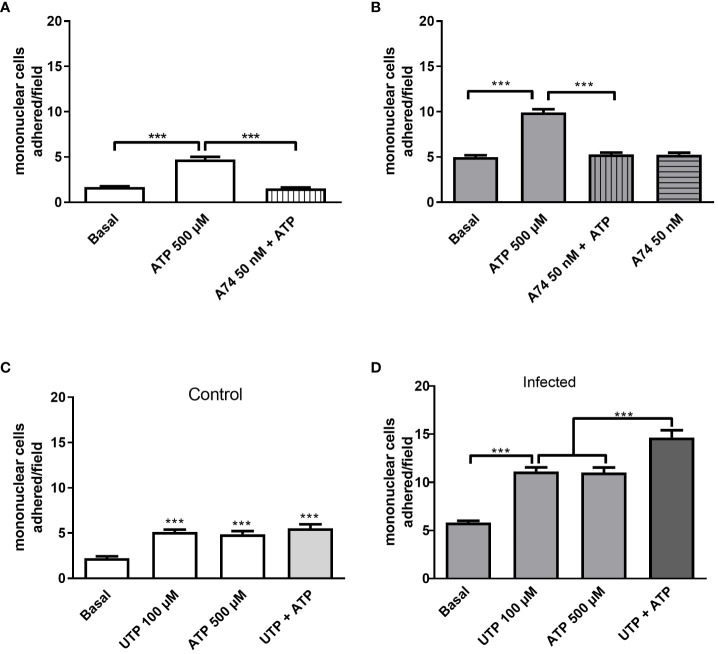
Evidence of endothelial P2Y_2_R and P2X7R cooperation increasing mononuclear cell adhesion to endothelial monolayer in the infected group. The selective P2X7R antagonist A740003 (50 nM, 30 min pretreatment) blocked 500 µM-induced mononuclear cell adhesion in control (**A**, white bars) and infected groups (**B**, gray bars). In the control group, we observed similar effects of UTP 100 µM (P2Y_2_R), ATP 500 µM (P2X7R), or the combination of both agonists **(C)**. In the infected group, P2Y_2_R and P2X7R coactivation with 100 µM UTP plus 500 µM ATP induced a higher mononuclear cell adhesion than each agonist alone **(D)**. Data were expressed as the mean and SEM of *n* independent cultures for each condition. ^***^ *p* < 0.001 (one-way ANOVA followed by Tukey’s multiple comparisons test, *n* = 3 or 4). A74, A740003 (selective P2X7R antagonist).

Previously, we showed that mesenteric endothelial cells express functional P2X7R, which is also known for its proinflammatory action (27). As ATP has a low affinity for mice P2X7R with EC_50_ values higher than 500 µM, we worked with a sub-maximal concentration of ATP (500 µM) (24). Accordingly, endothelial cell stimulation with 500 µM ATP for 10 min elicited monocyte adhesion in both groups, but the highest effect was observed in the infected group (**Figures 4A, B**). The selective P2X7R antagonist A740003 blocked the ATP effect, ruling out the contribution of another P2XR subtype (**Figure 4**).

Noteworthy, in the infected group, the coactivation of P2Y_2_R and P2X7R with UTP at 100 µM and ATP at 500 µM, respectively, induced an adhesion value higher than the effect of each agonist individually, suggesting a potential crosstalk between the signaling of both endothelial receptors in schistosomiasis (**Figures 4C, D**).

**Figure 5:**
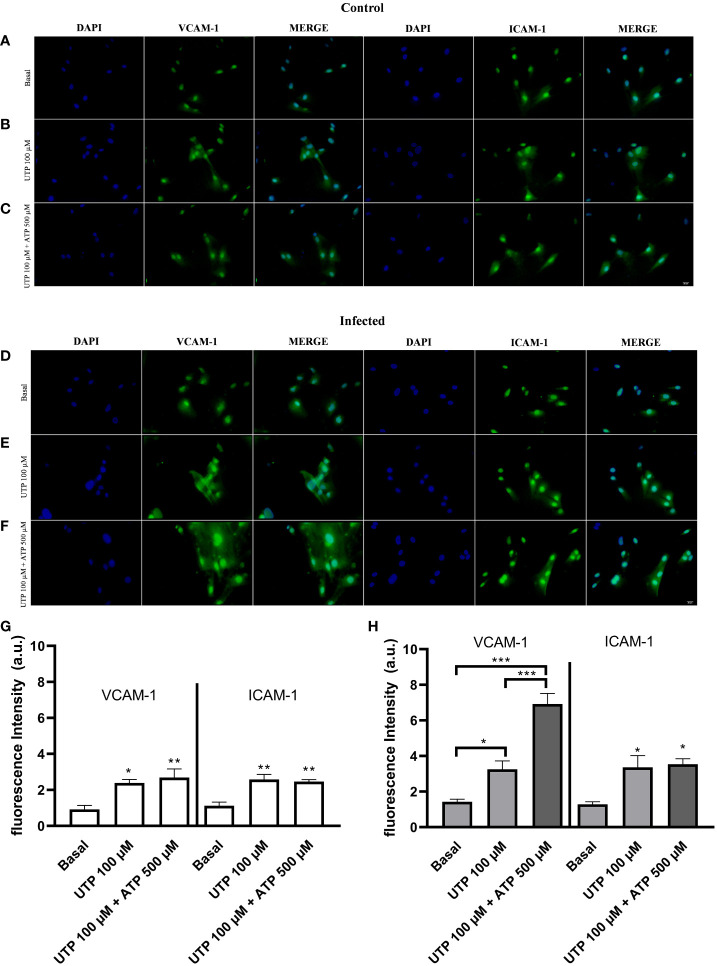
Endothelial VCAM-1 expression are selectively upregulated by the P2Y_2_R and P2X7R coactivation in the infected group. Immunocytochemistry staining of cultured endothelial cells using antibodies raised against VCAM-1 (green) or ICAM-1 (green) and nuclear fluorescence using DAPI (blue) (scale bar = 20 µm; ×400). Basal: endothelial cells were incubated with DMEM supplemented with 0.2% SFB for 5 h **(A**, **D)**. Endothelial cells were incubated with UTP 100 µM (5 h, **B**, **E**) or UTP 100 µM (5 h) plus ATP 500 µM (10 min) diluted in DMEM supplemented with 0.2% SFB **(C**, **F)**. Representative images from the control **(A**–**C)** and infected groups **(D**–**F)** were randomly chosen. Similar results were obtained in other experiments (*n* = 4 for each condition). The fluorescence intensity was quantified for the control (white bars, **G**) and infected groups (gray bars, **H)** and expressed as arbitrary units (a.u.). Data were expressed as the mean and SEM of *n* independent cultures for each condition. ^***^ *p* < 0.001, ^**^ *p* < 0.01, ^*^ *p* < 0.05 (one-way ANOVA followed by Tukey’s multiple comparisons test, *n* = 4) **(G, H)**.

**Figure 6:**
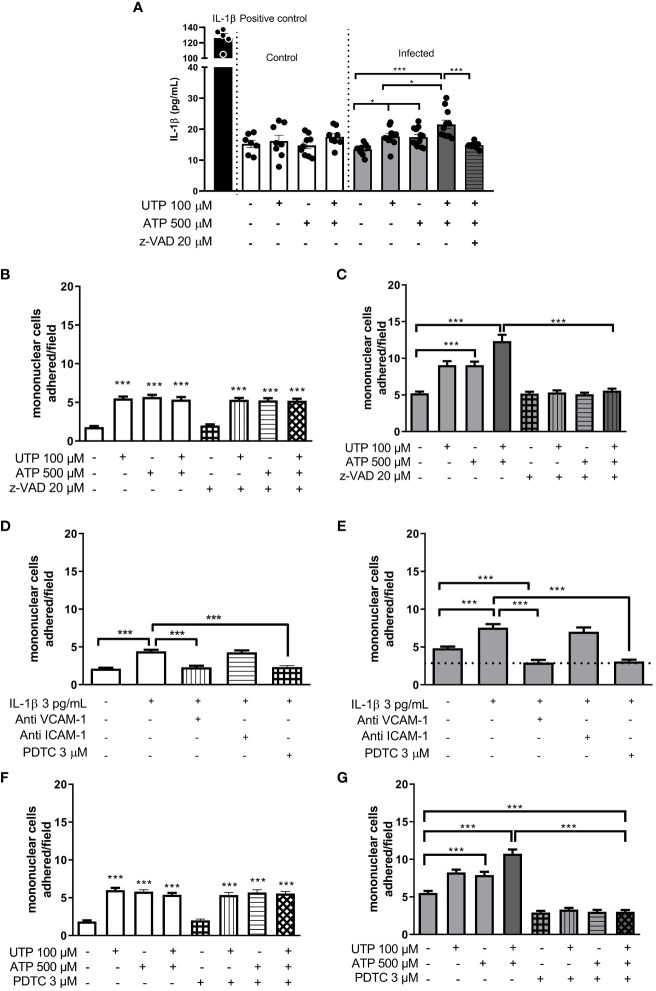
Evidence of endothelial P2Y_2_R and P2X7R cooperation increasing IL-1β release and mononuclear cell adhesion in the infected group. White bars, control; gray bars, infected group. P2Y_2_R and P2X7R activation with UTP 100 µM and ATP 500 µM, respectively, increased IL-1β release only in the infected group **(A)**. In this group, the coactivation of both P2 receptors induced a higher effect than the activation of each receptor individually **(A)**. The caspase inhibitor (Z-VAD-FMK 20 µM) blocked the UTP plus ATP-induced IL-1β release by endothelial cells **(A)** and the mononuclear cell adhesion only in the infected group **(B**, **C)**. The IL-1β-induced mononuclear cell adhesion was blocked by endothelial preincubation with anti-VCAM-1 antibody (dilution 1:50) or NF-κB inhibitor PDTC 3 µM, but not by anti-ICAM-1 antibody (dilution 1:50) **(D, E)**. Moreover, in the infected group, both the VCAM-1 antibody and PDTC also reduced basal values of mononuclear cell adhesion **(E)**. PDTC prevented the mononuclear cell adhesion induced by UTP, ATP, or UTP plus ATP only in the infected group **(F**, **G)**. Data were expressed as the mean and SEM of *n* independent cultures for each condition. ^***^ *p* < 0.001, ^*^ *p* < 0.05 (one-way ANOVA followed by Tukey’s multiple comparisons test, *n* = 3–6 **(A)**. ^***^ *p* < 0.001 (one-way ANOVA followed by Tukey’s multiple comparisons test, *n* = 3 (**B–G**).

**Figure 7:**
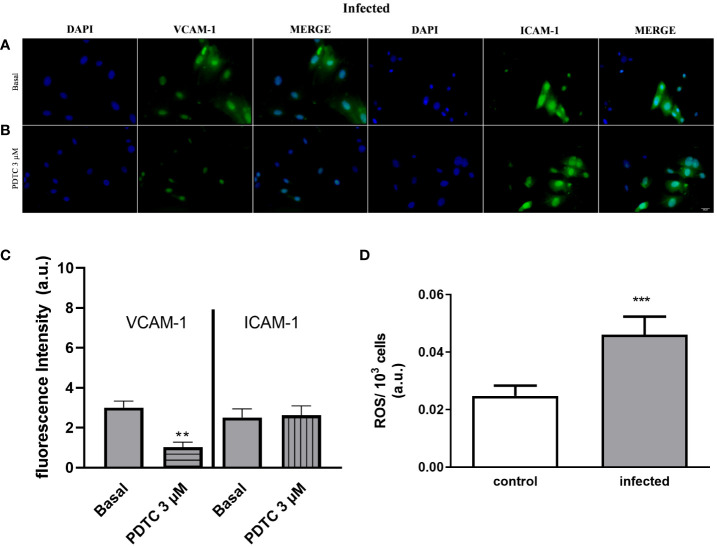
Selective endothelial VCAM-1 expression downregulation by the NF-κB inhibitor and increased endothelial ROS production in the infected group. Immunocytochemistry staining of cultured endothelial cells using antibodies raised against VCAM-1 (green) or ICAM-1 (green) and nuclear fluorescence using DAPI (blue) (scale bar = 20 µm; ×400). Basal: endothelial cells from the infected group were incubated with DMEM supplemented with 0.2% SFB for 30 min **(A)**. Endothelial cells from the infected group were incubated with PDTC 3 µM diluted in DMEM with 0.2% SFB for 30 min **(B)**. **(A, B)** Representative images of the infected group were randomly chosen. Similar results were obtained in different experiments (*n* = 4). The fluorescence intensity quantification was determined for the infected group (gray bars). Data were expressed as the mean and SEM of *n* independent cultures for each condition. ^**^ *p* < 0.01 (Student’s *t*-test, *n* = 4) **(C)**. White bar, control; gray bar, infected group. Endothelial cells produce a higher amount of ROS during schistosomiasis infection **(D)**. Mesenteric endothelial cells were incubated with nitro blue tetrazolium solution, which reacts with ROS (mainly superoxide anion) forming formazan crystals. Data were expressed as mean and SEM of *n* independent cultures for each condition. ^***^ *p* < 0.001 (Student’s *t*-test, *n* = 4).

Next, we investigated the molecular mechanisms involved in monocyte adhesion. Immunocytochemistry data revealed that the endothelial cell stimulation with UTP 100 µM significantly increased VCAM-1 and ICAM-1 expression in the control (**Figures 5B, G**) and in the infected groups (**Figures 5E, H**) compared to basal levels (**Figures 5A, D, G**) (*p* < 0.05). In the control group, the induction of VCAM-1 and ICAM-1 expressions by the costimulation with both agonists did not differ from the UTP condition (**Figures 5C, G**; *p* > 0.05). However, in the infected group, the coactivation with UTP at 100 µM and ATP at 500 µM selectively upregulated VCAM-1 expression compared to P2Y_2_ activation alone (**Figures 5E, F, H**; *p* < 0.001), suggesting a positive cooperation between both receptors during schistosomiasis. In good accordance, in this group, the VCAM-1 expression induced by the combination of both agonists was higher than in the control group (**Figures 5G, H**; *p* = 0.0014). Additionally, in the infected group, the coactivation of P2Y_2_R and P2X7R increased the release of IL-1β as compared to UTP or ATP individually (**Figure 6A**), which could be involved in endothelial cell activation. As P2X7R-mediated IL-1β release depends on inflammasome/caspase-1 pathways, we used Z-VAD-FMK as an inhibitor (**Figure 6A**) before assaying monocyte adhesion in response to purinergic agonists. In the control group, Z-VAD-FMK (20 µM) did not alter cell adhesion induced by 100 µM UTP, 500 µM ATP, or the combination of both agonists (**Figure 6B**). However, in the infected group, Z-VAD-FMK blocked the effects of both agonists, either isolated or in combination (**Figure 6C**). Furthermore, IL-1β (3 pg/mL) also increased monocyte adhesion in both groups, and this effect was inhibited by VCAM-1 blockage but not by ICAM-1 blockage (**Figures 6D, E**). Moreover, in the context of schistosomiasis, the anti-VCAM-1 blockage also reduced basal monocyte adhesion (**Figure 6E**; *p* < 0.01). The IL-1β-induced monocyte adhesion was inhibited by endothelial cell pretreatment with the nuclear factor (NF)-κB inhibitor PDTC (3 µM) (**Figures 6D, E**). Given that VCAM-1 contributed to P2Y_2_R-induced monocyte adhesion (**Figure 3F**) and its expression depends on NF-κB (28), we incubated endothelial cells with PDTC before assaying monocyte adhesion in response to purinergic agonists. PDTC only inhibited cell adhesion induced by 100 µM UTP, 500 µM ATP, or the combination of both agonists in the infected group (**Figures 6F, G**). In this group, PDTC also reduced basal monocyte adhesion (**Figure 6G**), consistent with the observations in **Figure 6E**. In good accordance with functional data, PDTC selectively inhibited endothelial VCAM-1 expression in the infected group (**Figures 7A–C**) without any effect in the control group (**Supplementary Figure S3**). Of note, PDTC pretreatment did not reduce ICAM-1 expression in both groups (**Figures 7A–C**; **Supplementary Figure S3**). Given that NF-κB inhibition by PDTC has previously been linked to an antioxidant-sensitive mechanism (28), we evaluated basal ROS production by endothelial cells. Our findings revealed that cells from the infected group released more ROS, mainly superoxide anion, than controls (**Figure 7D**).

## Discussion

The presence of Schistosoma parasites and eggs triggers host immune responses and damages the mesenteric blood vessels (19). Previously, we showed remarkable vasculitis, vascular fibrosis, and endothelial dysfunction in the murine model of schistosomiasis (10, 29). Endothelial cells show a noteworthy heterogeneity in health and disease (30), and cell damage is a hallmark mechanism of nucleotide release to the extracellular milieu, activating purinergic signaling. Current data using mesenteric endothelial cells primed by schistosomiasis unveiled positive cooperation between P2Y_2_R and P2X7R signaling, increasing IL-1β release, VCAM-1 expression, and monocyte adhesion. Therefore, the link between PAMP-driven endothelial P2Y_2_R/P2X7R signaling may play a critical role in mesenteric inflammation during schistosomiasis.

Schistosomiasis is endemic in many countries, and therefore people are infected chronically or constantly reinfected. There is only one WHO-approved drug with anti-schistosome activity but with partial cure rates and devoid of anti-inflammatory effects (6). This unmet need impairs precise medicine.

*S. mansoni* infection induces similar immune responses in mice and humans, therefore validating the preclinical murine model for schistosomiasis (3). Intravascular Schistosoma and eggs release PAMPs that prime endothelial cells to a proinflammatory/proadhesive phenotype *in vitro* (10, 31). Cytokines such as TNF-α, IL-1β, IL-6, IL-4, and IL-10, among others, show increased serum levels during schistosomiasis, reflecting the polarization and complexity between Th1 and Th2 immune responses (32, 33).

In the infected group, we observed greater monocyte adhesion in response to UTP than in controls. The selective P2Y_2_R antagonists ARC118925XX and suramin blocked the UTP effect in both groups, restoring basal values, and the UTP effect was not altered by CD39 inhibition, discarding a putative action of the metabolite UDP. In good accordance, it has been shown that the effect of UTP is equally blunted by suramin treatment, endothelial cell P2Y_2_R knockdown (34, 35), and global or endothelial cell-specific P2Y_2_R-deficient mice (21, 22). Moreover, P2Y_4_R, which is insensitive to suramin, did not play a role in this process (36). Altogether, we consider that P2Y_2_R mediated the endothelial effects of UTP. Furthermore, although the expression of members of the purinergic signaling may vary in pathological conditions (27, 31, 37), the endothelial expression of P2Y_2_R at the protein level is not altered at this stage of the disease. Regarding receptor signaling, both canonical and noncanonical P2Y_2_R signaling, classically linked to VEGF receptor transactivation (35), contributed to monocyte adhesion in both groups. Although inhibiting Src with SU6656 reduced UTP-induced monocyte adhesion, our data indicate that the noncanonical P2Y_2_R-Src-VEGF receptor signaling pathway remains unaffected. However, the functional outcome of the infection resulted in mesenteric endothelial cells, and the P2Y_2_R downstream signaling linked to intracellular Ca^2+^ in response to UTP seemed to be upregulated.

*In vivo*, a significant increase in the number of monocytes in the peritoneal cavity was observed compared to the control group. Considering that leukocyte adhesion precedes transmigration across the vascular wall, these *in vitro* data could support the *in vivo* findings. Accordingly, Stachon et al. (38) showed that in P2Y_2_R-competent mice, the intraperitoneal administration of ATP increases leukocyte counting in the peritoneal lavage. Of note, since P2Y_2_R activation increases monocyte adhesion (38–41), there is a growing interest in the role of endothelial P2Y_2_R signaling in endothelial dysfunction.

Previous studies highlighted the important role of endothelial Ca^2+^ mobilization in P2Y_2_R-induced intercellular adhesions (40, 41). According to fluorometric data using fura-2, endothelial cells from both groups showed a rapid and transient increase of intracellular Ca^2+^ concentration in response to P2Y_2_R activation. Moreover, the peak value and area under the curve in the infected group were higher than in the control. Even though those cells also express P2Y_1_R (activated by ADP) and P2X7R (activated by mM concentrations of ATP), they are not expected to be activated with this agonist concentration (27, 31, 42). Moreover, in the infected group, endothelial cell pretreatment with the inhibitor of intracellular Ca^2+^ signaling BAPTA-AM or the PLC inhibitor U73122 prevented the agonist effect and reduced basal leukocyte adhesion (*p* < 0.001), suggesting that endothelial cell Ca^2+^ homeostasis is altered by schistosomiasis.

In our model, UTP induced endothelial VCAM-1 expression. This adhesion molecule seems essential for leukocyte adhesion since the treatment of endothelial cells with anti-VCAM-1 monoclonal antibody prevented the proadhesive effect of UTP. Accordingly, P2Y_2_R^−/−^ mice lack the UTP-induced VCAM-1 expression and monocyte adhesion (21, 39, 43). Moreover, VCAM-1-dependent monocyte adhesion is abrogated in endothelial cells with reduced G_q/11_ protein expression (43). Therefore, the endothelial VCAM-1 expression is an essential partner of P2Y_2_R-Ca^2+^ signaling leading to monocyte adhesion and inflammation. Of note, VCAM-1 has been identified as a significant contributor to the pathogenesis of schistosomiasis (44–47), and endothelial cell treatment with an anti-VCAM-1 monoclonal antibody reduces the egg adhesion to these cells (47). In line with this, our current study revealed a higher level of immunoreactivity for VCAM-1 in endothelial cells from the infected group compared to the control group. Endothelial ICAM-1 inhibition also prevented UTP-mediated monocyte adhesion in both groups, but with similar effects.

Mesenteric endothelial cells also express P2X7R (27), which, once activated, exhibit remarkable versatility such as increasing intracellular Ca^2+^, activating inflammasome, and releasing inflammatory mediators (16, 27). Therefore, we investigated if those endothelial receptors could also be engaged in monocyte adhesion. The endogenous low-affinity agonist ATP (500 µM) induced intercellular adhesions in both groups, and its effect was blocked by the pretreatment of endothelial cells with the selective P2X7R antagonist A740003.

P2Y_2_R and P2X7R have been linked to IL-1β release in a caspase-1-dependent way during infectious diseases (48). *In vivo* treatment of mice with apyrase reduced schistosomiasis-induced liver inflammation without altering parasitic load, suggesting that purinergic signaling is relevant for pathology (49). Interestingly, endothelial P2X7R expression is reduced during chronic schistosomiasis, but their role in inflammation has not been addressed (27). Noteworthy, in murine osteoblasts, P2Y_2_R and P2X7R are expressed in caveola, and caveolin (Cav)-1 knockdown upregulates P2X7R signaling (50). Moreover, recent findings showed that P2Y_2_R and P2X7R receptors cooperate, in which P2Y_2_R activation alters the kinetics of P2X7R, favoring its activation (51). However, whether this cooperation also occurs in endothelial cells and/or if they have any role in infectious diseases remains elusive.

In the current work, the activation of P2Y_2_R and P2X7R with UTP (100 µM) and ATP (500 µM), respectively, induced IL-1β release only by endothelial cells from the infected group, i.e., primed cells, which is in good accordance with the knowledge that P2 receptors are the second signal to inflammasome activation (16, 52). Moreover, these primed cells are hyperresponsive to the coactivation of P2Y_2_R and P2X7R, releasing more IL-1β and mediating an increased VCAM-1 expression and leukocyte adhesion than in response to each receptor alone. Altogether, these data suggest the occurrence of a positive cooperation between P2Y_2_R and P2X7R and inflammasome response, despite the reduced expression of P2X7R. In the infected group, caspase-1 inhibition (Z-VAD-FMK) abolished the effect of both agonists, either individually or in combination, suggesting that the cytokine could be involved in monocyte adhesion. In good accordance, endothelial cell treatment with IL-1β induced monocyte adhesion, which was blunted by the pretreatment with anti-VCAM-1 but not by anti-ICAM-1 antibody. On the contrary, in the control group, endothelial caspase-1 inhibition did not affect the effect of the agonists, which corroborated the lack of effect of UTP and ATP on IL-1β release in the absence of cell priming. An interesting finding was that in the infected group, the inhibition of endothelial NF-κB signaling by PDTC not only abolished the effects of the agonists (individually or in combination) but also reduced IL-1β-mediated and basal mononuclear cell adhesion, unveiling a downstream P2Y_2_R/P2X7R-IL-1β-NF-κB-VCAM-1 signaling in schistosomiasis. In good accordance, previous data showed that PDTC inhibits VCAM-1, but not ICAM-1, expression (28) and highlighted the role of VCAM-1 in schistosomiasis pathogenesis (46, 47).

Given the roles of ROS and Ca^2+^ in leukocyte adhesion (53), their involvement in P2X7R signaling (52), and the fact that PDTC inhibits NF-κB in a redox-sensitive way and reduces basal monocyte adhesion, we measured basal endothelial ROS production. We found increased levels of ROS (mainly superoxide anion), suggesting that endothelial NF-κB signaling is upregulated in schistosomiasis.

Schistosomiasis shifts the delicate balance between pro- and anti-inflammatory signaling due to various virulence factors released by worms and eggs. This altered immunological landscape can have profound consequences. It can lead to the development of chronic inflammation, tissue damage, and the formation of granulomas. Previously, we described an up-regulation of P2Y_1_R-dependent leukocyte adhesion during schistosomiasis (31). On the other hand, endothelial P2Y_6_R did not contribute to mononuclear cell adhesion in the infected group (*unpublished data*). Therefore, it seems that schistosomiasis’s pathophysiology involves selective members of the vascular purinergic signaling pathway (54). As leukocyte adhesion to the endothelial monolayer precedes transmigration (55), current data may partially explain the peritonitis (10, 56) and vascular inflammation (19) observed in schistosomiasis.

A previous *S. mansoni* infection boosts the host’s immune response against eggs in subsequent infections (57). Current evidence shows that eggs (and their antigen Smp40) reduce endothelial Cav-1 expression, disrupting endoprotective signaling, leading to dysfunctional phenotype of human and mouse endothelial cells and lung injury (58). Noteworthy, given that Cav-1 knockdown upregulates P2X7R signaling (50), it is possible that in our model, endothelial Cav-1 signaling reduction upregulates P2X7R signaling and inflammasome activation counteracting the reduced receptor expression (27). Moreover, eggs alter gut microbiota in both quantitative and qualitative ways (58). This underscores the pivotal role of the host’s gut microbiota in the crosstalk between schistosomes and host immunity, highlighting the possibility of host dysbiosis as a third partner in the disease process (59). Notably, gut dysbiosis contributes to the vascular inflammation of mesenteric venules (60), and recent evidence linking gut dysbiosis and alterations in purinergic signaling has emerged (61). One limitation of the present work is that we did not evaluate schistosomal-induced gut dysbiosis to make any correlation. Therefore, further experiments are welcome to explore the putative link between schistosomiasis, gut dysbiosis, and the development of mesenteric inflammation induced by P2 receptors.

**Figure 8:**
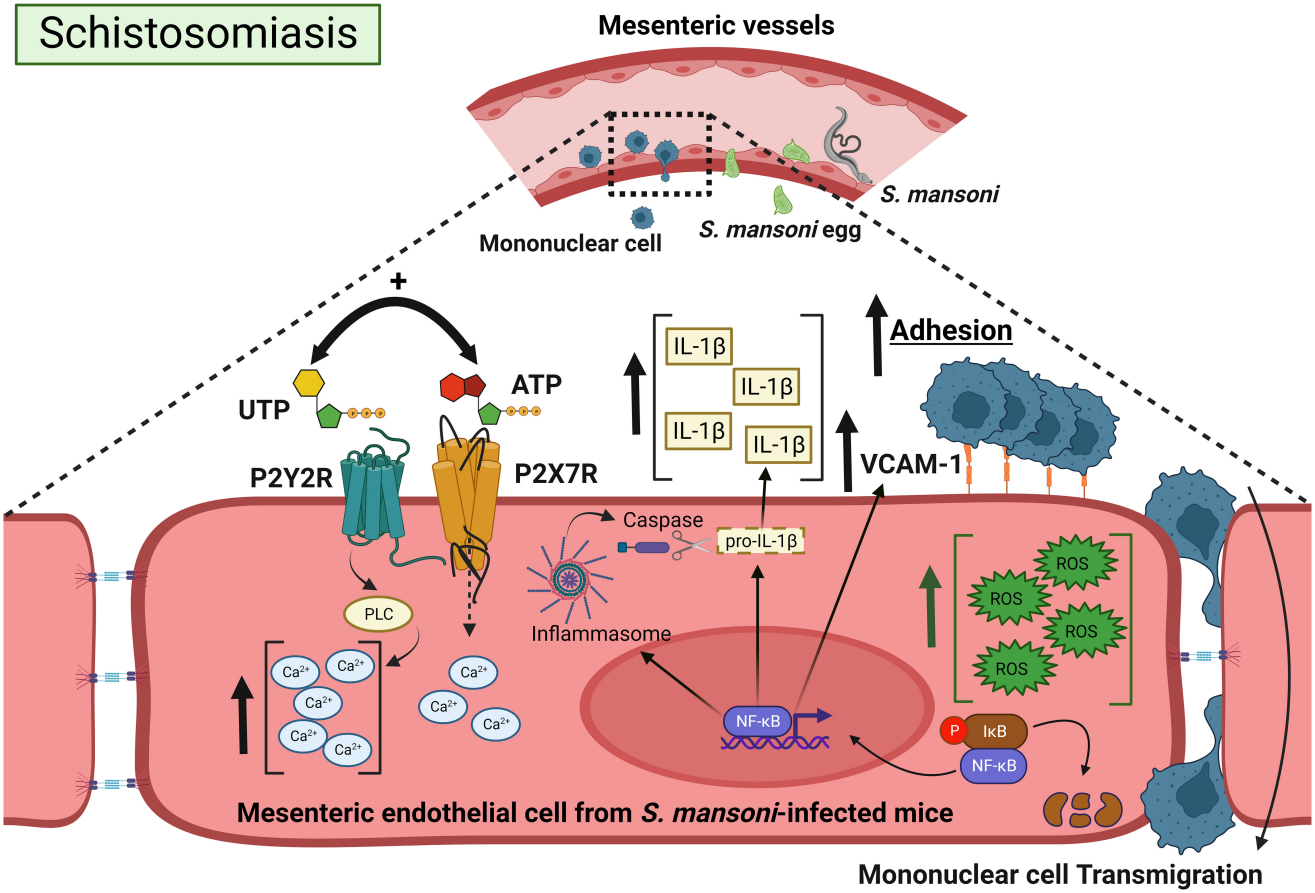
Positive cooperation between endothelial P2Y_2_R and P2X7R signaling favoring mononuclear cell adhesion during schistosomiasis. Schistosoma eggs adhere to endothelial cells and extravasate to the peritoneum or intestinal lumen, triggering a granulomatous response. The coactivation of endothelial P2Y_2_R-P2X7Rs increases IL-1β release and VCAM-1 expression. The increased endothelial Ca^2+^ canonical signaling together with ROS promotes NF-κB signaling, favoring IL-1β release, VCAM-1 expression, mononuclear cell adhesion, and transmigration. Created with BioRender.com.

In conclusion, current data unveiled a positive cooperation between endothelial P2Y_2_R and P2X7R signaling favoring mononuclear cell adhesion, which involved IL-1β release, NF-κB signaling, and VCAM-1 expression (**Figure 8**). These findings raise the possibility that PAMP-induced dysfunctional P2Y_2_R-P2X7R signaling contributes to mesenteric inflammation during schistosomiasis. These discoveries advance our understanding of schistosomiasis and unveil a putative pharmacological target to reduce morbidity.

## Data availability statement

The raw data supporting the conclusions of this article will be made available by the authors, without undue reservation.

## Ethics statement

The animal study was approved by Ethics Committee of the Federal University of Rio de Janeiro (CEUA, A1/19-048-16 and 124/22). The study was conducted in accordance with the local legislation and institutional requirements.

## Author contributions

NO: Conceptualization, Formal analysis, Investigation, Visualization, Writing – original draft, Writing – review & editing. MM: Formal analysis, Investigation, Writing – original draft. NM: Formal analysis, Investigation, Writing – original draft. AT: Investigation, Writing – original draft, Formal analysis. LP: Investigation, Writing – original draft. LC: Formal analysis, Investigation, Writing – original draft. RC-S: Funding acquisition, Writing – review & editing, Resources. LS: Formal analysis, Funding acquisition, Writing – review & editing, Resources. CS: Conceptualization, Formal analysis, Funding acquisition, Project administration, Visualization, Writing – original draft, Writing – review & editing, Resources, Supervision.

## Supplementary material

Supplementary Figure 1 The P2Y_2_R antagonist suramin inhibited UTP-mediated mononuclear cell adhesion to endothelial cells. Mesenteric endothelial cells were pre-incubated with suramin 50 μM for 30 min before stimulation with UTP (100 μM) stimulus in both groups **(A, B)**. Data were expressed as mean and SEM. *** p < 0.001 (one-way ANOVA followed by Tukey's multiple comparisons test, n = 3 different cultures for each condition) Sur = suramin.

Supplementary Figure 2 Inhibition of the endothelial Src-VEGF pathway prevented the pro-adhesive effect of P2Y_2_R. Mesenteric endothelial cells were pre-incubated with non-canonical signaling P2Y_2_R inhibitor (SU6656 5 μM, 30 min pre-treatment) before UTP 100 μM stimulus in both groups **(A, B)**. Data were expressed as mean and SEM, n = 3 individual experiments with different animals for each condition *** p <0.001 (one-way ANOVA followed by Tukey's multiple comparisons test). SU6656 = SrC inhibitor.

Supplementary Figure 3 The NF-κB inhibition did not alter the endothelial VCAM-1 and ICAM-1 expressions in the control group. Immunocytochemistry staining of cultured endothelial cells using antibodies raised against VCAM-1 (green) or ICAM-1 (green), and nuclear fluorescence using DAPI (blue) (scale bar = 20 µm, x400). Basal: endothelial cells were incubated with DMEM supplemented with SFB 0.2% for 30 min **(A)**. Endothelial cells were incubated with PDTC 3 μM (NF-κB inhibitor) diluted in DMEM supplemented with SFB 0.2% for 30 min **(B)**. Representative images from control group were randomly chosen. Similar results were observed in other experiments (n=4). The fluorescence intensity was determined for Control group = white bars. Data were expressed as mean and SEM of n independent cultures for each condition, n = 4) **(C)**.
