# Supplementary figures and images for: P2Y2-P2X7 receptors cross-talk in primed mesenteric endothelial cells upregulates NF-κB signaling favoring mononuclear cell adhesion in schistosomiasis
Source: Front Immunol. 2024 Jan 4;14:1328897. doi: 10.3389/fimmu.2023.1328897 (PMC10794548; doi:10.3389/fimmu.2023.1328897)

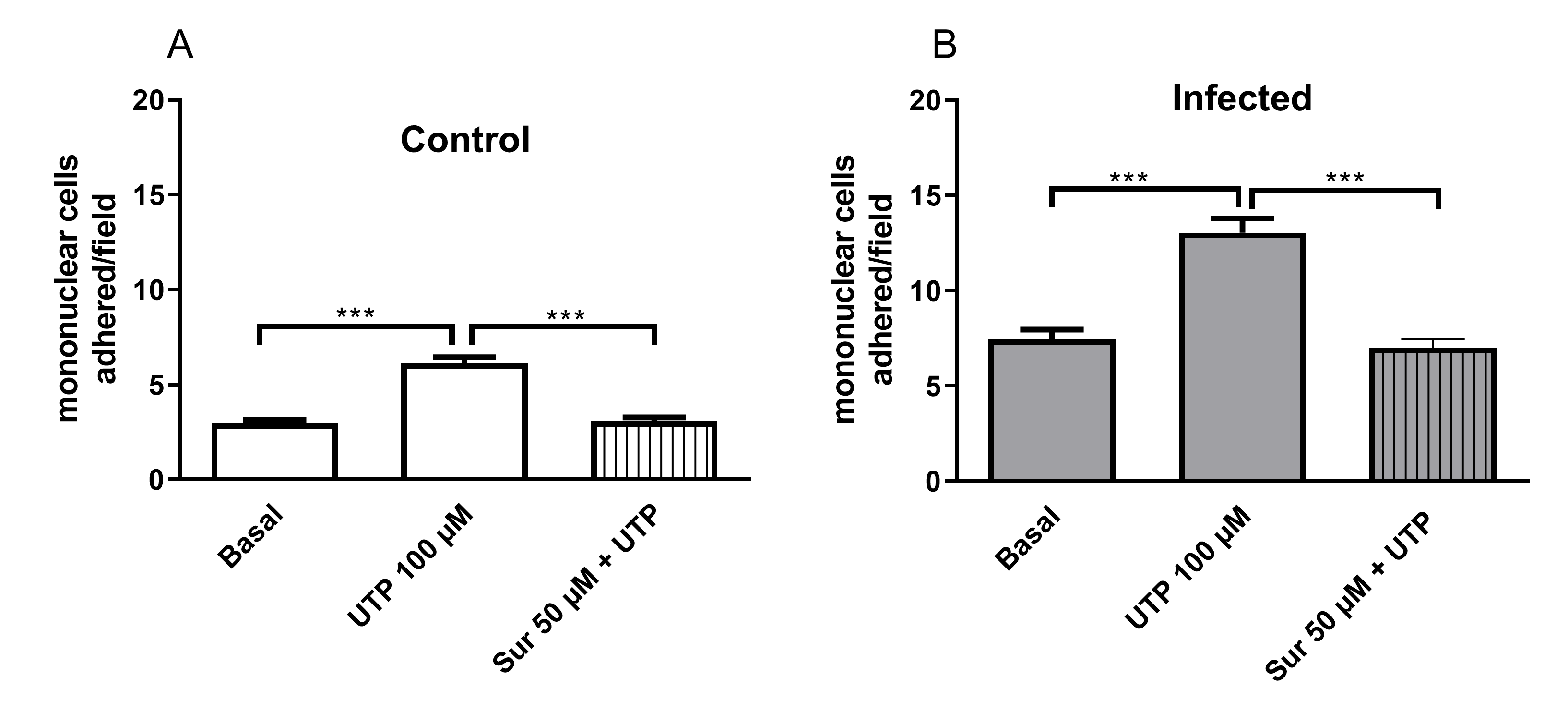

Supplement: Supplementary Figure 1 — The P2Y2R antagonist suramin inhibited UTP-mediated mononuclear cell adhesion to endothelial cells. Mesenteric endothelial cells were pre-incubated with suramin 50 μM for 30 min before stimulation with UTP (100 μM) stimulus in both groups (A, B). Data were expressed as mean and SEM. *** p < 0.001 (one-way ANOVA followed by Tukey's multiple comparisons test, n = 3 different cultures for each condition) Sur = suramin. [file Image_1.tif]

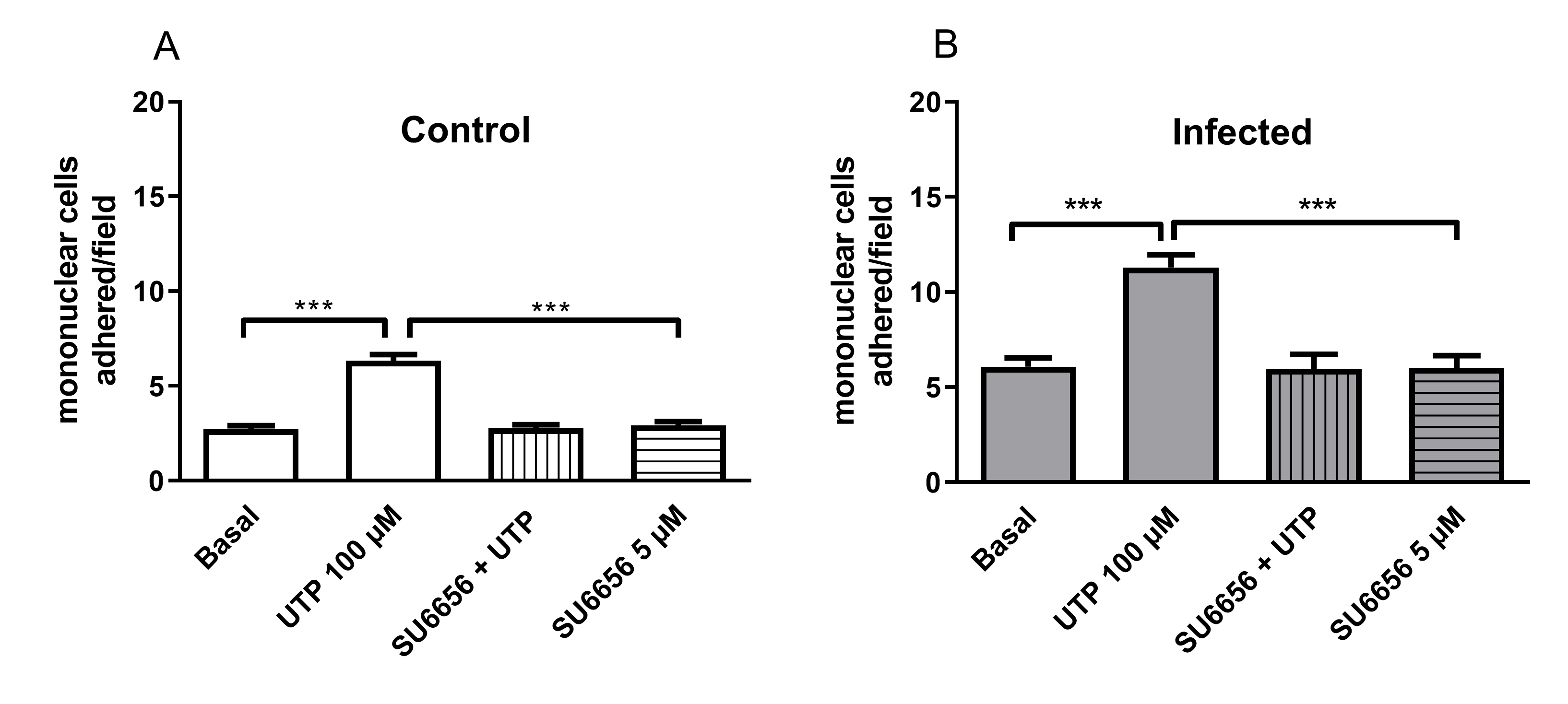

Supplement: Supplementary Figure 2 — Inhibition of the endothelial Src-VEGF pathway prevented the pro-adhesive effect of P2Y2R. Mesenteric endothelial cells were pre-incubated with non-canonical signaling P2Y2R inhibitor (SU6656 5 μM, 30 min pre-treatment) before UTP 100 μM stimulus in both groups (A, B). Data were expressed as mean and SEM, n = 3 individual experiments with different animals for each condition *** p <0.001 (one-way ANOVA followed by Tukey's multiple comparisons test). SU6656 = SrC inhibitor. [file Image_2.tif]

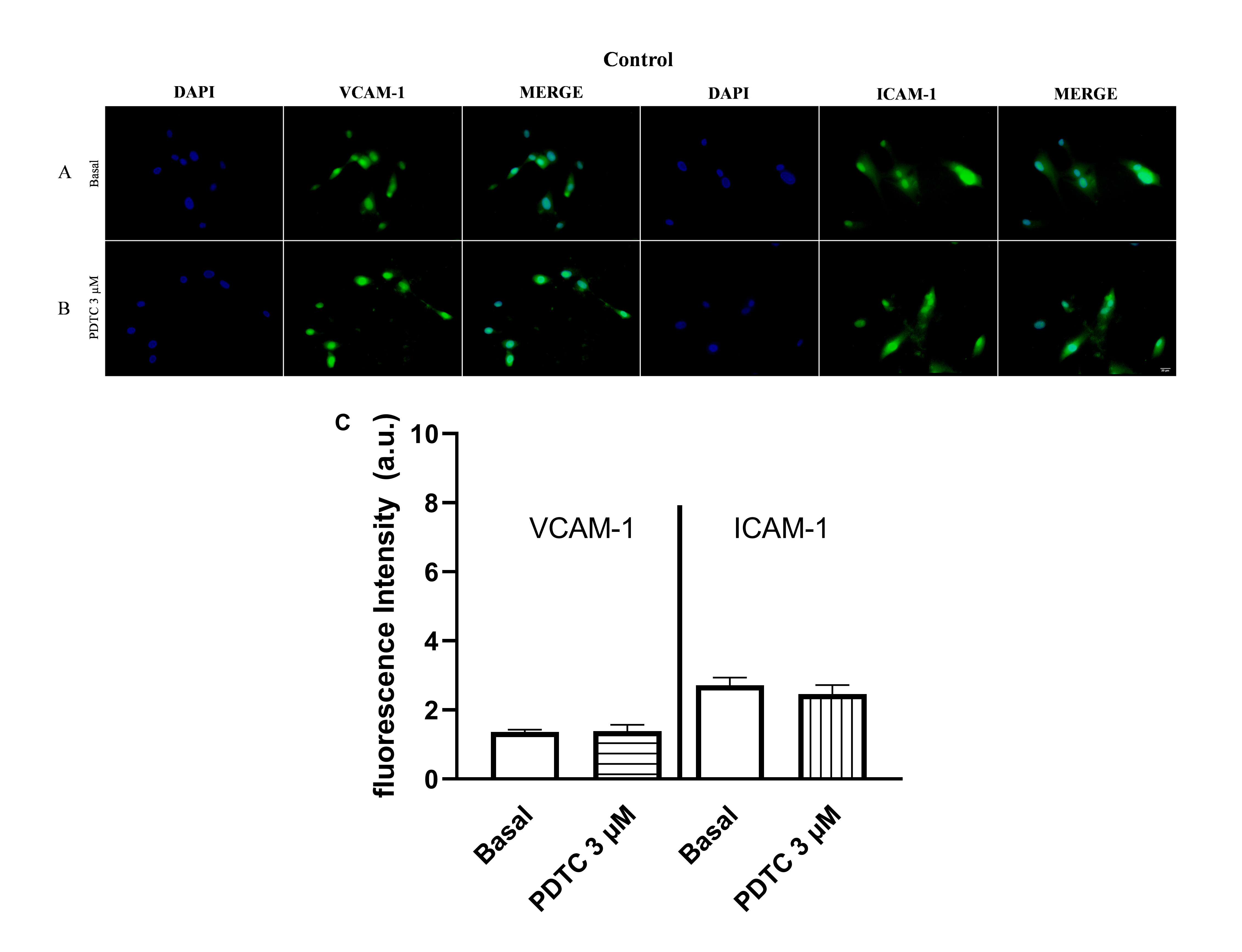

Supplement: Supplementary Figure 3 — The NF-κB inhibition did not alter the endothelial VCAM-1 and ICAM-1 expressions in the control group. Immunocytochemistry staining of cultured endothelial cells using antibodies raised against VCAM-1 (green) or ICAM-1 (green), and nuclear fluorescence using DAPI (blue) (scale bar = 20 µm, x400). Basal: endothelial cells were incubated with DMEM supplemented with SFB 0.2% for 30 min (A). Endothelial cells were incubated with PDTC 3 μM (NF-κB inhibitor) diluted in DMEM supplemented with SFB 0.2% for 30 min (B). Representative images from control group were randomly chosen. Similar results were observed in other experiments (n=4). The fluorescence intensity was determined for Control group = white bars. Data were expressed as mean and SEM of n independent cultures for each condition, n = 4) (C). [file Image_3.tif]
